# COMPARING SOCIO-ECONOMIC CONDITIONS OF MOTHER AND CHILDREN WITH LEPROSY IN ENDEMIC AND NON-ENDEMIC AREAS IN EAST JAVA, INDONESIA

**DOI:** 10.21010/ajid.v15i2.9

**Published:** 2021-03-18

**Authors:** Flora Ramona Sigit Prakoeswa, Ghina Shabrina Awanis, Aini Azizah, Budi Prasetyo, Santi Martini, Hardyanto Soebono, Dominicus Husada, Hari Basuki Notobroto, Muhammad Yulianto Listiawan, Anang Endaryanto, Cita Rosita Sigit Prakoeswa

**Affiliations:** 1 Doctoral Program, Faculty of Medicine, Airlangga University, Indonesia.Department of Dermatology and Venereology, Faculty of Medicine, Universitas Muhammadiyah Surakarta, Indonesia; 2 Faculty of Medicine, Universitas Sebelas Maret, Indonesia; 3 Master of Public Health Program, Faculty of Medicine, Public Health, and Nursing, Universitas Gadjah Mada; 4 Department of Obstetrics and Gynecology, Faculty of Medicine, Airlangga University / Dr. Soetomo General Academic Hospital, Surabaya, Indonesia; 5 Faculty of Public Health, Airlangga University, Indonesia; 6 Department of Dermatology and Venereology, Faculty of Medicine, Public Health and Nursing, Gadjah Mada University, Indonesia; 7 Department of Pediatric, Faculty of Medicine, Airlangga University / Dr. Soetomo General Academic Hospital, Surabaya, Indonesia; 8 Department of Dermatology and Venereology, Faculty of Medicine, Airlangga University / Dr. Soetomo General Academic Hospital, Surabaya, Indonesia

**Keywords:** leprosy, mother and child, endemic and non-endemic, socioeconomic

## Abstract

**Background:**

Leprosy is a disease that causes social, psychological, and economic issues. Failure to treat the causes of the immune system dysregulation in endemic areas of leprosy conditions makes the transmission of the bacteria easier. This paper aims to analyze the comparison of family income, occupation types of mothers and fathers, number of children, access to health facilities, and education of mothers, fathers, and children in mothers and children with leprosy in endemic and non-endemic areas.

**Materials and Methods:**

A cross sectional study by survey was done in both an endemic and a non-endemic area of leprosy in Tuban Regency, East Java, Indonesia. Retrieval of research data was done using interview techniques. Respondents who participated in this study were 106 pairs of mother and child respondents who met the research restriction criteria. Subjects were divided into 5groups based on diagnosis of leprosy and area of living. Bivariate analysis was performed by comparing the independent variables in each group A, B, C, and D with group E.

**Results:**

It was found that the variables that differed significantly between the endemic and non-endemic areas were the variable number of children with a p-value=0.004, family income with a p-value=0.049 and the variable mother’s education with a p-value=0.016. Meanwhile, other variables do not have significant difference.

**Conclusions:**

We found significant difference on the number of children, father’s education, mother’s education, and family income. These variables can be a risk factor for leprosy. To make efforts to prevent the transmission of leprosy, stakeholders should consider these factors.

## Introduction

Leprosy is a contagious skin disease caused by *Mycobacterium leprae*. Leprosy is known as ‘The Great Imitator Disease’ because this disease is often not easily recognised because it has symptoms that are almost similar to other skin diseases. This is also caused by the leprosy bacteria itself undergoing a long division process of 2–3 weeks and an incubation period of 2–5 years or more (Health Ministry of Indonesia, 2018). Leprosy is thought to have originated in India, 600 BC. In 1873, Armauer G. Hansen in Norway managed to identify the organisms that cause leprosy, so that this disease is also known as Hansen’s disease (World Health Organization, 2017).

Leprosy is a disease that is considered negative by society because of the ulceration, mutilation and the deformity it causes, which causes social, psychological, and economic problems. There are two types of leprosy, namely Paucibacillary (PB) and Multibacillary (MB) (Gunawan *et al.*, 2018). Leprosy is transmitted through direct contact through the skin and respiratory tract repeatedly and over a long period (Astutik and Kiptiyah, 2016). The low level of knowledge can also cause leprosy because some of them think that this leprosy is a curse from God, a hereditary disease, or because of some occult cause that is difficult to cure. It is considered shameful and causes disgrace to the family. The impact of this disease is that people tend to have a negative attitude towards leprosy patients, such as rejecting, shunning, looking down on, and criticizing them. This stigma reflects that the level of public knowledge about leprosy is still low (Pescarini *et al.*, 2018).

The incidence of leprosy in the world in 2016 based on WHO data has increased, from 211,973 in 2015 to 214,783 in 2016. As much as 94% of the leprosy incidents were reported by 14 countries with more than 1000 new cases each year (World Health Organization, 2017). This shows that there are still many areas that are the pockets of high endemicity of leprosy in the world. Southeast Asia is a region with the highest incidence of leprosy, with 161,263 cases in 2016. Indonesia is a country with the 3rd highest leprosy incidence contributor in the world, with 16,286 cases, after Brazil (25,218 cases) & India (145,485 cases) (World Health Organization, 2017).

East Java is the province with the highest incidence of leprosy on the island of Java, with 3,373 cases and the second-highest level of leprosy disability cases, with 293 cases last year 2017 (Health Ministry of Indonesia, 2018). East Java has nine leprosy endemic cities/districts where the spread of this disease has not been eliminated, one of them is Tuban Regency (The Department of Health of East Java Province, 2019).

Previous study in Indonesia found that women with leprosy faced more discrimination than men and also suffer stigma linked to marriage (van Brakel et al., 2012). Women’s health, especially those of childbearing age, may affect the immune dysfunction of their children if a woman has experienced infection, malnutrition, obesity, exposure to cigarette smoke during pregnancy (PrabhuDas *et al.*, 2015). Failure to treat the causes of the immune system dysregulation in populations living in endemic leprosy conditions makes the transmission of bacteria easier as the host becomes more vulnerable to leprosy (Sadhu and Mitra, 2018). The dominant position of women in the care of their families increases the risk of women with leprosy to spread the disease to other household members, particularly their children. Moreover, women in developing countries tend to receive late-term health care coverage for any health-related issues (Sarkar and Pradhan, 2016).

In addition, epidemiological studies of leprosy in children can point out the important aspects of the environment that influence the leprosy transmission in an endemic area; because children have lower mobility than adults (Adriaty *et al.*, 2020). Evidence also suggests that the degree of vulnerability of the individual, the extent of exposure, and associated environmental factors could potentially influence the transmission. Complete understanding of ecological and environmental components may unfold the gaps in knowledge regarding the mode of transmission of leprosy (Joshua *et al.*, 2016). Hence, it is necessary to analyse the socioeconomic conditions in mother and children in particular. It is also important to compare the conditions between an endemic and non-endemic area since there are many different ecological and environmental components at play in the transmission of leprosy.

Leprosy is not a disease that causes instant death, like other infectious diseases. However, leprosy is a chronic infectious disease with complex problems, ranging from medical problems to social and economic problems. Poverty, population density, slum settlements, can be factors that trigger the transmission of leprosy because if the surrounding population becomes denser, the chances of contracting leprosy will also increase (Vieira *et al.*, 2018). Leprosy is closely related to the socio-economic conditions of the community, which are classified as middle to lower class. Besides that, the low level of knowledge which has the possibility of contracting leprosy, and poor personal hygiene conditions are also triggering factors for the spread of leprosy (Nery et al., 2019). Family income and occupation are closely related to family welfare. Family income in particular has a positive correlation with number of people in a house, that also poses a major risk factor because of the increased household density (Rahmah *et al.*, 2018). In the case of leprosy, existing evidence suggests that poor living conditions may be associated with increased risk (Pescarini *et al.*, 2018).

Although there have been many studies on social factors related to leprosy, the discussion has focused more on the problem of poverty. Evaluation of the specific social conditions regarding leprosy is still lacking (Nery *et al.*, 2019).

This study aims to analyze the comparison of family income, occupation types of mothers and fathers, the number of children, access to health facilities, and education of mothers, fathers, and children of leprosy patients with mothers and children in endemic and non-endemic areas.

## Materials and Methods

### Study Design and Data Collection

This cross-sectional study was conducted in endemic area and non-endemic area of leprosy in Tuban Regency, East Java Province from March to June 2020. Tuban Regency is considered a leprosy pocket area with 173 cases in 2018, of which 43.35% cases were maternal leprosy and the leprosy cases among children accounted for 5.81% of the total cases.

Study population of this study include mother and her child who lives in endemic and non-endemic area. The inclusion criteria for subjects with leprosy was those with confirmed diagnosis of leprosy and aged between 5-18 years old for children; whilst the excluded were those with any leprosy reaction, poor general condition, and diagnosed with inflammatory or autoimmune disorder, allergy, or infection other than leprosy, and pregnancy. All of the subjects gave informed consent. Leprosy cases were selected from the local primary health center’s registry data. Thereafter, to confirm the diagnosis, the subjects underwent clinical examination done by a dermatologist and then acid-fast staining by trained health and laboratory professional from Dr. Soetomo General Hospital and Tropical Disease Centre of Airlangga University.

**Figure 1:**
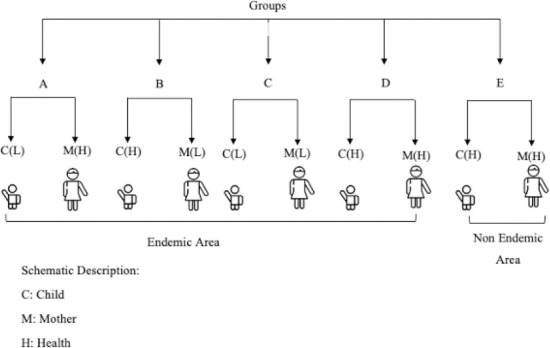
Scheme of The Grouping Method

Subjects were divided into 5 groups as follows: group A was child with leprosy and mother without leprosy in an endemic area; group B was child without leprosy and mother with leprosy in an endemic area; group C were child with leprosy and mother with leprosy in an endemic area; group D was child without leprosy and mother without leprosy in an endemic area; group E was child without leprosy and mother without leprosy in non-endemic area. This grouping method is explained in **Figure 1**. The data analysis technique is done by doing data reduction, data coding, and analysis. This study has received an Ethical Clearance from the Health Research Ethics Committee of Dr. Soetomo Surabaya with number 1664 / KEPK / XI / 2019.

### Statistical analysis

Statistical analysis used in the research is univariate and bivariate analysis. In univariate analysis, the frequency and percentage of respondents for each variable is displayed. Bivariate analysis was performed by comparing the independent variables in each group A, B, C, and D with group E. The statistical analysis used was the Pearson Chi-Square test. Next is an analysis comparing the total group A, B, C, and D (all respondents in endemic areas) with group E (respondents in non-endemic areas). The statistical analysis used was the ANOVA (analysis of variance) test. ANOVA test is a test used to test the mean difference between the ABCD and E groups. Meanwhile, the variables that do not have a normal distribution were tested by Mann-whitney test. All significant p-values were calculated based on a 2-sided statistical test, with a significance level of α = 0.05.

## Results

Respondents who participated in this study were 106 pairs of mother and child respondents who met the research restriction criteria. The research data were taken directly at the respondent’s house using a questionnaire sheet.

There are groups of respondents studied, namely groups A, B, C, D, and group E. Groups A to D live in leprosy endemic areas, while group E has homes in non-endemic areas of leprosy. Demographic characteristics of the research subjects studied included diagnosis, gender, education, number of children, education, income, and also access to health facilities. Univariate analysis was carried out by analysing the percentage of each of these variables as a whole, in groups A, B, C, D, and E. These demographic characteristics can be seen in Table 1.

**Table 1:** Characteristic of Research Subjects.

Variable	Whole (%)	A (%)	B (%)	C (%)	D (%)	E (%)
Mother’s Diagnosis						
Healthy	74.5	100.0	0.0	0.0	100.0	100.0
Leprosy	25.5	0.0	100.0	100.0	0.0	0.0

Child Diagnosis						
Healthy	79.2	0.0	100.0	0.0	100.0	100.0
Leprosy	20.8	100.0	0.0	100.0	0.0	0.0

Gender of Children						
Male	48.1	73.7	37.5	66.7	39.4	48.1
Female	51.9	26.3	62.5	33.3	60.6	51.9

Mother’s Education						
No school	4.7	10.5	4.2	0.0	6.1	0.0
Not completed in primary school	5.7	10.5	4.2	0.0	3.0	7.4
Graduated from elementary school	45.3	63.2	62.5	100.0	27.3	33.3
Graduated from junior high school	32.1	10.5	29.2	0.0	51.5	29.0
Graduated from high school	7.5	0.0	0.0	0.0	6.1	22.0
College	4.7	5.3	0.0	0.0	6.1	7.4

Father’s Education						
No school	4.7	15.8	0.0	33.3	3.0	0.0
Not completed in primary school	7.5	10.5	12.5	0.0	3.0	7.4
Graduated from elementary school	52.8	57.9	66.7	66.7	48.5	40.7
Graduated from junior high school	17.9	5.3	8.3	0.0	21.2	33.3
Graduated from high school	13.2	0.0	12.5	0.0	18.2	11.1
College	3.8	10.5	0.0	0.0	6.1	7.4

Children’s Education						

No school	12.3	10.5	20.8	0.0	12.1	14.8
Not completed in primary school	26.4	36.8	20.8	0.0	27.3	44.4
Graduated from elementary school	29.2	31.6	25.0	0.0	36.4	22.2
Graduated from junior high school	15.1	21.1	12.5	0.0	9.1	14.8
Graduated from high school	17.0	0.0	20.8	100.0	15.2	3.7
College	0.0	0.0	0.0	0.0	0.0	0.0

Number of children						
1	24.5	0.0	16.7	0.0	24.2	51.9
2	55.7	63.2	54.2	66.7	63.6	40.7
3	15.1	26.3	25.0	33.3	9.1	3.7
4	2.8	5.3	4.2	0.0	3.0	0.0
5	1.9	5.3	0.0	0.0	0.0	3.7

Father’s occupation						
Does not work	9.4	10.5	4.2	33.3	9.1	11.1
entrepreneur	21.7	10.5	20.8	0.0	3.0	33.3
Farmer	30.2	31.6	41.7	33.3	21.2	18.5
Fisherman	3.8	5.3	4.2	33.3	30.3	3.7
Labor	25.5	31.6	25.0	0.0	27.3	22.2
Civil servants	0.9	0.0	0.0	0.0	0.0	0.0
Others	8.5	10.5	4.2	0.0	9.1	11.1

Mother’s occupation						
Does not work	45.3	26.3	37.5	33.3	57.6	51.9
entrepreneur	14.2	15.8	8.3	0.0	15.2	18.5
Farmer	17.0	31.6	20.8	33.3	12.1	7.4
Fisherman	0	0.0	0.0	0.0	0.0	0.0
Labor	17.9	26.3	33.3	33.3	9.1	7.4
Civil servants	2.8	0.0	0.0	0.0	3.0	7.4
Others	2.8	0.0	0.0	0.0	3.0	7.4

Income						
0 - 2,500,000	81.1	84.2	91.7	66.7	78.8	74.1
2,500,000 - 5,000,000	14.2	10.5	4.2	33.3	15.2	22.2
> 5,000,000	4.7	5.3	4.2	0.0	6.1	3.7

Access to health facilities for mothers	100.0	100.0	100.0	100.0	100.0	100.0
Have	0.0	0.0	0.0	0.0	0.0	0.0
Not Have						

Access to children’s health facilities						
Have	100.0	100.0	100.0	100.0	100.0	100.0
Not Have	0.0	0.0	0.0	0.0	0.0	0.0

Group A: Child with leprosy and mother without leprosy in an endemic area, group B: child without leprosy and mother with leprosy in an endemic area, group C: child with leprosy and mother with leprosy in an endemic area, group D: child without leprosy and mother without leprosy in an endemic area, group E: child without leprosy and mother without leprosy in non-endemic area.

The majority of child respondents were female (51.9%). The majority of mothers and fathers graduated from elementary school (45.3% and 52.8%). The majority of the respondent’s family is from the middle to lower class, with the majority of the family income is less than IDR 2,500,000.00.

In Table 2 it can be seen from all the variables studied, the variables that have differences, between groups in endemic areas and groups in non-endemic areas, are the number of children and the education of the father. Variables other than that do not have a significant difference.

**Table 2:** Analysis of Differences between Groups A, B, C, D and Group E.

Variable	A: E	B: E	C: E	D: E	(A + B + C + D): E.
Number of children	P = 0.001 *	P = 0.025 *	P = 0.192	P = 0.171	P = 0.004 *

Family income	P = 0.500	P = 0.222	P = 0.878	P = 0.974	P = 0.049*

Mother’s education	P = 0.046 *	P = 0.056	P = 0.287	P = 0.217	P = 0.016 *

Children’s education	P = 0.396	P = 0.058	P = 0.735	P = 0.360	P = 0.734

Father’s education	P = 0.062	P = 0.115	P = 0.048 *	P = 0.709	P = 0.113

Mother’s job	P = 0.083	P = 0.304	p = 0.543	P = 0.657	P = 0.427

Father’s occupation	P = 0.950	P = 0.357	P = 0.283	P = 0.795	P = 0.730
Access to children’s health facilities	p = .a	p = .a	p = .a	p = .a	p = .a

Access to maternal health facilities	p = .a	p = .a	p = .a	p = .a	p = .a

Group A: Child with leprosy and mother without leprosy in an endemic area, group B: child without leprosy and mother with leprosy in an endemic area, group C: child with leprosy and mother with leprosy in an endemic area, group D: child without leprosy and mother without leprosy in an endemic area, group E: child without leprosy and mother without leprosy in non-endemic area.

There is a difference between groups A and B and group E on the variable number of children. The difference in the number of children in groups A and E has a significance value of p = 0.001 (p <0.05), while in groups B and E it has a significance value of p = 0.025 (p <0.05). In groups C and D, p-value > 0.05, which means there is no significant difference between groups C and D and group E.

In the family income variable, both in groups A, B, C, and D, there is no difference with group E because the p-value of the four groups was more than 0.05 (p> 0.05). There is no difference between groups A, B, C, and D and group E.

Education of mother variable shows a difference between groups A and E with a value of p = 0.046 (p <0.05). However, in groups B, C, and D, there is no difference with group E. In the father’s education variable, the different group from group E is group D, with a value of p = 0,048 (P<0.05). Children’s education variables do not differ across groups.

Access to health facilities cannot be determined because of the constant data. All mother and child respondents in groups A, B, C, D, and E have access to health facilities.

After conducting a comparative analysis of each group A, B, C, and D against group E, a comparative analysis was carried out for all groups A, B, C, and D in endemic areas with Group E in non-endemic areas. Based on the results of the ANOVA test, it was found that the variables that differed significantly between the endemic and non-endemic areas were the variable number of children with a p-value = 0.004, family income with a p-value = 0.049 and the variable mother’s education with a p-value = 0.016. Meanwhile, other variables do not have a significant difference.

## Discussion

Family income is closely related to family welfare. Family income in groups A, B, C, and D has a significant difference with group E. This is consistent with a previous study in Indonesia, where correlation between family income and leprosy risk is studied in Kediri, East Java. Family income has a positive correlation with number of people in a house, that also poses as a major risk factor for leprosy as in household contacts (Rahmah *et al.*, 2018). According to a study in Brazil, it was found that low income can increase the risk of getting leprosy by 40% (Nery *et al.*, 2019). Another study in Northeastern Brazil confirmed the same finding. Persons with no income have positive correlation with leprosy risk (p-value = 0.01) as well as poor people (p-value = 0.001) (de Sousa *et al.*, 2020).

The types of occupations of the respondents in endemic and non-endemic areas vary among the following: entrepreneurs, farmers, laborers, fishermen, and civil servants. There is no significant difference between the respondents in endemic and non-endemic areas. According to Pescarini (2018), manual labour is one of the risk factors for leprosy. This is in agreement with the work of Munstasir *et al*. (2018), who reported high-risk work related to the incidence of leprosy in Kupang, NTT.

There is a difference between the variable number of children in endemic and non-endemic areas with a value of p = 0.004. The number of children in a family is related to the density of individuals in a house. The more the number of children, the more the number of family members increases and can increase the contact between family members. Overcrowded houses can increase contact between sufferers and people who are still health (Pescarini *et al.*, 2018).

Research conducted in leprosy endemic areas also shows similar results. Home contact is also associated with leprosy with a value of p = 0.006. In this study, it is stated that residential areas with middle to lower economic levels tend to have this risk (de Sousa *et al.*, 2020). Apart from affecting household contact, the large number of children can also cause density which has an impact on high humidity in the house, which can affect the occurrence of leprosy (Rahmah *et al.*, 2018).

Observational research on school children also found that overcrowding of houses is a risk factor for leprosy. The density of the house can increase the contact between healthy children and people with leprosy (Pedrosa *et al.*, 2018; Vieira *et al.*, 2018). Reducing the risk of a contact history of leprosy can be done, but depends on preventive measures such as immunoprophylaxis treatment and index cases (Gomes *et al.*, 2019).

In this study, all respondents have access to health service facilities so that no conclusions can be drawn. However, access to health services is known to have a relationship with the incidence of leprosy, as described in the study in Bangkalan. In that study, it was stated that the time to travel to health services was related to the incidence of leprosy with a value of p = 0.000 (Nurcahyati and Wibowo, 2016). However, access to health services is not only measured by travel time. Other factors can affect access to health services, namely stigma. It is necessary to provide information related to leprosy and also to reduce the stigma against leprosy patients to increase patient motivation to seek treatment. In a study in Brazil, although access to travel time and transportation could be overcome, 45.1% of patients considered their symptoms not serious so they were reluctant to seek treatment. 42.6% of patients had cases of misdiagnosis that resulted in late treatment; and 48.4% of patients visited doctors for fear of worsening symptoms. This is also exacerbated by the presence of a stigma that prevents patients from seeking treatment (Henry *et al.*, 2016).

The education of fathers and mothers in endemic areas is different from that of fathers and mothers in non-endemic areas. Meanwhile, there is no difference in children’s education. This is because children’s education is now more evenly distributed. Besides, there is a 9-year compulsory education policy that causes no difference between the variables of children’s education in endemic and non-endemic areas.

Father’s education can affect the incidence of disease in family members, especially leprosy. Based on research conducted in Brazil, the level of education can increase the risk of leprosy. Not only the education level of the patient but also the level of education of the head of the household, namely the father. The low level of education of fathers can increase the risk of disease by 2 times compared to the high level of education of fathers. Low level of education of fathers can be a risk of leprosy in children in the family (Nery *et al.*, 2019).

Research in Kediri, East Java, also agreed with the effect of education on leprosy. Higher education levels can have an indirect effect on preventing leprosy. Higher education is associated with higher levels of family income and personal hygiene for each family member. Both of these are risk factors for leprosy in Kediri. This means, indirectly, education can also influence the occurrence of leprosy (Rahmah *et al.*, 2018).

A low level of education can be a risk factor for leprosy. Individuals who do not go to school after they finish elementary school are at a level of education that is prone to contracting leprosy. Low levels of education mean that disease transmission continues. This can cause an area to remain an endemic area for leprosy (de Sousa *et al.*, 2020).

Mothers play a key role in a family. Generally, it is the mother who maintains the survival of the family. Mothers who have a low level of knowledge will also have a low impact on the condition of their families. The factors that influence the level of knowledge are education, occupation, social, environmental, belief, age, social, cultural, and economic. The higher the level of knowledge, the higher the individual’s ability to assess a material (Garamina, 2017). Besides, low levels of knowledge can also lead to low levels of hygiene and healthy living habits and low personal hygiene, and this can certainly lead to leprosy (Pescarini *et al.*, 2018).

## Conclusion

This study found differences among the number of children, father’s education, and maternal education among respondents in endemic and non-endemic areas. These variables can be a risk factor for leprosy. To make efforts to prevent the transmission of leprosy, stakeholders should consider these factors. Based on these results, it can be said that the eradication of leprosy is not only the responsibility of health workers, but it requires the collaboration of various related parties to jointly tackle leprosy, especially in Tuban Regency. Further studies need to be conducted to analyze other socioeconomic markers in endemic and non-endemic areas that are related to leprosy.

**List of abbreviations:**
PB: - paucibacillary
MB: – multibacillary
WHO: - World Health Organization
ANOVA: - analysis of variance
